# The active role of the transcription factor Sp1 in NFATc2-mediated gene regulation in pancreatic cancer

**DOI:** 10.1186/s12858-019-0105-4

**Published:** 2019-01-29

**Authors:** Manuela Malsy, Bernhard Graf, Katrin Almstedt

**Affiliations:** 1 Department of Anesthesiology, University Medical Center, Regensburg, Franz Josef Strauss Allee 11, 93053 Regensburg, Germany; 2 Department of Obstetrics and Gynecology, University Hospital, Mainz, Mainz, Germany

**Keywords:** Sp1, NFATc2, Pancreatic cancer, Binding partner, Cancer, C-terminus, N-terminus, Proliferation

## Abstract

**Background:**

Adenocarcinoma of the pancreas is one of the most aggressive tumor diseases affecting the human body. The oncogenic potential of pancreatic cancer is mainly characterized by extremely rapid growth triggered by the activation of oncogenic signaling cascades, which suggests a change in the regulation of important transcription factors. Amongst others, NFAT transcription factors are assumed to play a central role in the carcinogenesis of pancreatic cancer. Recent research has shown the importance of the transcription factor Sp1 in the transcriptional activity of NFATc2 in pancreatic cancer. However, the role of the interaction between these two binding partners remains unclear. The current study investigated the role of Sp1 proteins in the expression of NFATc2 target genes and identified new target genes and their function in cells. A further objective was the domain of the Sp1 protein that mediates interaction with NFATc2.

The involvement of Sp1 proteins in NFATc2 target genes was shown by means of a gene expression profile analysis, and the results were confirmed by quantitative RT-PCR. The functional impact of this interaction was shown in a thymidine incorporation assay. A second objective was the physical interaction between NFATc2 and different Sp1 deletion mutants that was investigated by means of immunoprecipitation.

**Results:**

In pancreatic cancer, the proto-oncogene c-Fos, the tumor necrosis factor TNF-alpha, and the adhesion molecule integrin beta-3 are target genes of the interaction between Sp1 and NFATc2. Loss of just one transcription factor inhibits oncogenic complex formation and expression of cell cycle-regulating genes, thus verifiably decreasing the carcinogenic effect. The current study also showed the interaction between the transcription factor NFATc2 and the N-terminal domain of Sp1 in pancreatic cancer cells. Sp1 increases the activity of NFATc2 in the NFAT-responsive promoter.

**Conclusions:**

The regulation of gene promotors during transcription is a rather complex process because of the involvement of many proteins that – as transcription factors or co-factors – regulate promotor activity as required and control cell function. NFATc2 and Sp1 seem to play a key role in the progression of pancreatic cancer.

**Electronic supplementary material:**

The online version of this article (10.1186/s12858-019-0105-4) contains supplementary material, which is available to authorized users.

## Background

Malignant tumor diseases are one of the major causes of death worldwide [1]. One tumor disease with a very low survival rate is adenocarcinoma of the pancreas [2]. Because of the lack of characteristic early symptoms and effective screening tests, most tumors are classified as incurable cancer with a poor prognosis at the time of diagnosis [3]. Thus, current research is focused on the development of alternative treatment methods that aim at the efficient modulation of specific signaling and transcription pathways [4]. The prerequisite for developing new therapeutic approaches to ‚targeted therapies’ is detailed knowledge on the carcinogenesis of pancreatic cancer [5].

The pathogenesis of pancreatic cancer shows characteristic changes in morphology that are associated with typical genetic alterations [6]. On the basis of this knowledge, a tumor progression model was developed describing the gradual process of pancreatic cancer from its neoplastic preliminary stage – termed PanIN (pancreatic intraepithal neoplasia) – to its malignant stage [7]. This process not only involves various mutations but also genetic changes such as oncogene mutations, changes in tumor suppressor genes, as well as over-expression of growth factors and their receptors [8]. This series of mutations upsets the balance between tumor-suppressing and tumor-promoting pathways.

The carcinogenesis of pancreatic cancer is characterized by the modified activation of signaling pathways and the changed regulation of important transcription factors [9], particularly of the family of NFAT transcription factors [10]. Buchholz et al. have shown the increasing synthesization of both NFATc1 and NFATc2 in the preliminary stages of pancreatic carcinoma [11]. Furthermore, immunohistochemically examined pancreatic tumor cells have shown expression of NFAT in 91.7% of cases. In more than 80% of pancreatic tumor tissue, such over-expression is caused by the amplification of NFATc2 on the chromosome 20q13 [12].

In pancreatic cancer, the oncogenic transcription factor Sp1 (specificity protein 1) plays a central role in the transcriptional and functional activity of NFATc2. Sp1 belongs to the family of zinc finger proteins. Sp1 is composed of an N-terminus consisting of activating domains that weight about 70 kDa as well as of 3 zinc fingers measuring about 15 kDa that are localized at the C-terminal part. The 3 zinc fingers simultaneously represent the DNA-binding domain [13]. The corresponding complex formation between Sp1 and its binding partners occurs at the intracellular level [14]. Santini et al. already described the interaction between NFATc2 and Sp1 in keratinocytes in 2001 [15]. This interaction could be confirmed by our research team for pancreatic cancer cells, in which transcription factors of the same immune complex directly interact at the NFAT-DNA target sequence GGAAA and also have a joint function [14]. However, the role of this interaction in pancreatic cancer is still unclear.

The current study investigated the role of Sp1 proteins in the expression of NFATc2 target genes and identified new target genes and their function in cells. Thus, a selective and therefore specific blockade of this oncogenic complex formation in the sense of ‘targeted therapy’ seems possible at a translational level. The domain of the Sp1 protein in which the interaction with NFATc2 takes place is of vital importance in this context to avoid disturbance of the physiological function of NFATc2 and Sp1 in the organism.

## Methods

Chemicals, reagents, equipment an methodology used in the current study were mostly described previously in [13].

### Cell lines

The human pancreatic adenocarcinoma cell lines PaTu 8988 t were obtained from H. P. Elsässer (Philipps University of Marburg, Germany). PaTu 8988 t cells were maintained in Dulbecco’s modified Eagle’s medium (Sigma-Aldrich) supplemented with 10% fetal calf serum (Sigma-Aldrich) and 1% Normocyn (Fa. Amaxa biosystems). Cells were cultured at 37 °C in humidified CO_2_ atmosphere (5%) and maintained in monolayer culture. Experiments were done with cells at ~ 70–80% confluence.

### Reagents, siRNA transfection, and transient transfection

Ionomycin was purchased from Sigma-Aldrich. For siRNA transfection, NFATc2 siRNA (5`-CCAUUAAACAGGAGCAGAAtt-3`), Sp1 siRNA (5`-GGUAGCUCUAAGUUUUGAUtt-3`) and the Silencer Negative Control were obtained from Ambion (Applied Biosystems). Cells were transfected with the siLentFect lipid reagent (Biorad) for 24 h according to the manufacturer’s protocol.

For the transient transfection of expression constructs, PaTu 8988 t cells were transfected 24 h after seeding at 70% density, using TransFast (Promega) as a transfection reagent according to the manufacturer’s instructions. The Sp1 ZF- flag and Sp1 N-term-flag expression constructs were kindly provided by Dr. J. S. Zhang (Mayo Clinic, Minnesota, USA), mNFATc2-HA by Dr. A. Rao (Harvard Medical School, Boston, USA), and the promoter constructs cisNFAT-Luc by Stratagene Garden Grove.

### Quantitative real-time polymerase chain reaction analysis and gene expression analysis

RNA was extracted using the RNeasy Mini Kit (Qiagen). First-strand complementary DNA was synthesized using a First Strand cDNA Synthesis Kit (SuperArray Bioscience Corporation). Quantitative reverse transcription-polymerase chain reaction (RT-PCR) analysis was done with 7500 Fast Real-time PCR and the SYBR Green PCR Master Mix Kit (Applied Biosystems) according to the manufacturer’s instructions. RPLP0 was used as a housekeeping gene for normalizing gene expression. Primers with the following sequences were used for expression analysis: cFos (forward: 5`-AGTCCTTACCTCTTCCGGAGATG-3`; reverse: 5`-GCCTGGCTCAACATGCTAC TAA-3`). All primers had been obtained from Biomers.

For analysis of gene expression according to the manufacturer’s instructions. Extracted mRNA is transcribed into cDNA and mixed with the ‘SuperArray SYBR Green Mastermix’ containing oligonucleotide primers followed by quantitative Real-Time-PCR analysis and evaluation by means of the ΔΔCT method. All primers had been obtained from Qiagen. The gene using listed in Additional file 1: Table S1.

### Subcellular fractionation, co-immunoprecipitation, and immunoblotting

For subcellular fractionation, cells were washed twice with cold DPBS and resuspended in lysis buffer (12.5 mL 1 M HEPES, ph 7.5, 7.5 mL 5 M NaCl, 1.25 mL 200 mM EGTA, 25 mL 100% Gycerin, 2.5 mL Triton X-100, 1.05 g NaF, 1.11 g Na4P2O7 × 10 H_2_O) containing protease inhibitors. After sonification, cells were centrifuged at 13.000 rpm for 5 min, and supernatants were transferred to new cups and incubated on ice.

For co-immunoprecipitation, 500 μg of lysates was immunoprecipitated with 4 μL of the indicated antibodies and protein G or A agarose (Roche Diagnostics). The immunoprecipitates were subjected to immunoblotting.

For Western blotting, protein extracts were analyzed by SDS-PAGE and blotted onto nitrocellulose. Upon protein extraction and gel transfer, membranes were washed in TBS washing buffer and incubated with peroxidase-conjugated secondary antibodies. Immunoreactive proteins were visualized by means of an enhanced chemiluminescence detection system (Western Blotting Detection Reagent, GE Healthcare). Membranes were probed with NFATc2 (Santa Cruz Biotechnology) and Anti-FLAG (Sigma-Aldrich) antibodies.

### Luciferase reporter assay

Cells were seeded onto 12 well plates; after 24 h, cells were either transfected with the indicated constructs or treated. Luciferase activity was measured with the Lumat LB 9501 (Berthold Technologies) luminometer and the dual Luciferase Reporter Assay System (Promega) according to the manufacturer’s instructions. Firefly luciferase values were normalized to Renilla luciferase activity and are shown as mean values ±SD.

### Proliferation assays

Cells were seeded onto 24 well plates and cultured in medium containing 10% FCS. 19 h after the indicated treatment with siRNA or transfection, [3H]thymidine (0.5 μCi/well) was added during the last 5 h of incubation. The cells were washed with 5% trichloroacetic acid, and the acid-insoluble fraction was dissolved using incubation in 1 mol/L NaOH at 37 °C for 30 min. Radioactivity was evaluated with a scintillation counter (Pharmacia).

### Statistical analysis

Data are presented as mean ± SD. The t-test was used for statistical evaluation of the data. *P* values of < 0.05 were considered statistically significant.

## Results

### Involve of Sp1 on gene transcription mediated by NFATc2

An expression profile of PaTu 8988 t cells was created to investigate the role of Sp1 proteins in the expression of NFATc2 target genes and to identify new target genes. A prerequisite for achieving this aim is the reliable translocation of NFATc2 into the cell nucleus by means of a stimulus. A suitable stimulant in this respect is Ionomycin that initiates the influx of calcium into the cell, which subsequently activates the calcium-calcineurin-NFAT signaling pathway, dephosphorylizes NFAT, dislocates the cell nucleus, and increases DNA affinity.

For this purpose, mRNA was extracted from various pre-treated PaTu 8988 t lysates:

1) Untreated control,

2) Cells treated with Ionomycin 1 h previously,

3) Cells, in which Sp1 is repressed with RNA interference, and

4) Cells, stimulated with siRNA for Sp1 and additionally with Ionomycin for 1 h.

**Fig. 1:**
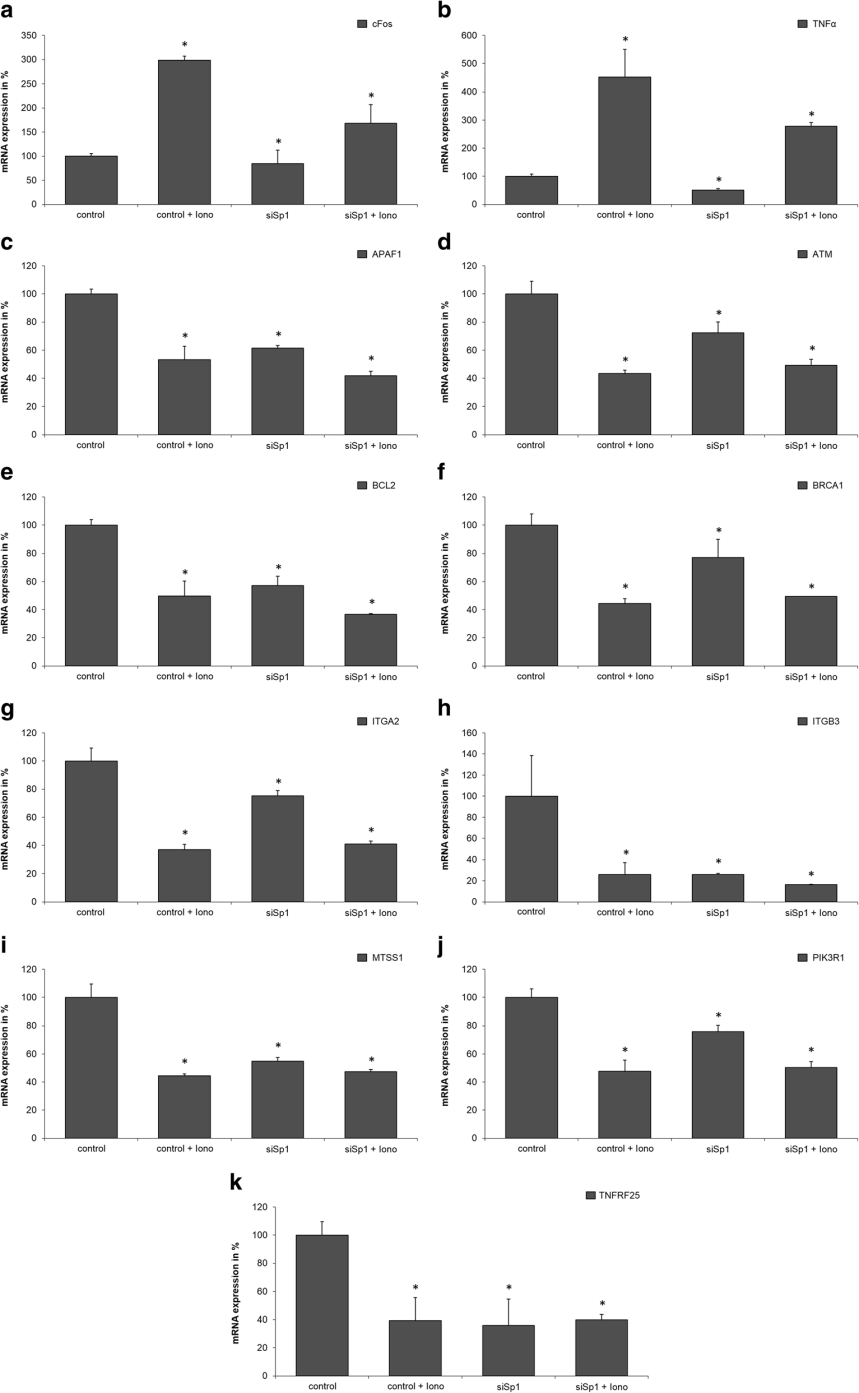
**a** - **k**: Expression profile analysis depending on the treatment with Ionomycin. Four different groups of mRNA are produced from PaTu 8988t lysates: 1) Untreated control lysates, 2) Lysates from cells treated with Ionomycin 1 h previously, 3) Cell lysates in which Sp1 is repressed with RNA interference, and 4) Lysates stimulated with siRNA for Sp1 and additionally with Ionomycin for 1 h. The lysates were assessed in the expression profile analysis that contained 89 different genes involved in the carcinogenesis of pancreatic cancer. After the administration of Ionomycin, altogether 11 genes (Fig. 1**a** - **k**) were expressed. The basal activity of the genes in the control lysate is normalized to the value of 100, and the influence of the regulatory change is shown as the x-fold increase or decrease of this control. Data are presented as mean ± SD. The t-test was used for statistical evaluation of the data. *P* values of <0.05 were considered statistically significant

The lysates were assessed in the expression profile analysis that contained 89 different genes involved in the carcinogenesis of pancreatic cancer. Expression was measured with the 7500 fast real time PCR system. The genes used for this purpose mainly stemmed from the following areas: cell cycle, transcription, signal transduction, and extracellular matrix. For analysis of gene expression the gene using listed in Additional file 1: Table S1. After the administration of Ionomycin, altogether 11 genes were expressed, for instance the proto-oncogene c-Fos and the tumor necrosis factor TNF-alpha that were both up-regulated (Fig. 1a + b). The results showed reduced synthesization of the molecules APAF1, ATM, BCL2, BRCA1, and TNFRSF25 involved in apoptosis, of the adhesion molecules integrin alpha-2 and integrin beta-3, of the metastasis suppressor gene MTSS1, as well as of phosphoinositide-3-kinase PI3K **(**Fig. 1c-k**)**.

It was remarkable that Sp1 inhibition minimized the effect of Ionomycin on c-Fos and TNF-alpha, whereas the administration of Ionomycin increased expression in the control group (band 2 in each case). Repression of Sp1 with RNAi technology significantly influenced the effect of Ionomycin by Sp1 inhibition (band 4 in each case). NFATc2 and Sp1 also had an antagonizing effect on ITGB3. Stimulation of control cells with Ionomycin decreased mRNA expression. The same effect could be achieved by inhibiting Sp1 with RNAi technology. However, treatment of cells with a combination of siSp1 and Ionomycin decreased mRNA expression by further 25% **(**Fig. 1h**)**. According to our results, Sp1 only plays a tangential role in regulating NFATc2 in the 8 remaining genes.

### Quantitative RT-PCR for confirming the expression profile

**Fig. 2:**
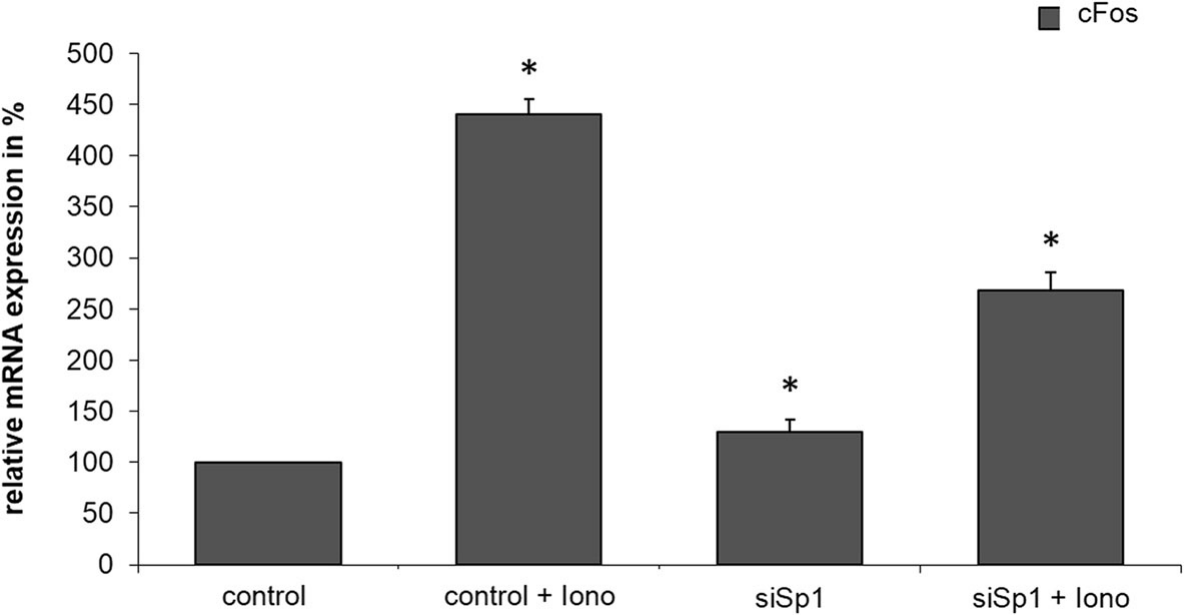
Quantitative Real Time-PCR analysis of 8988 t cells of c-Fos. PaTu 8988 t cells are divided into four groups followed by isolation of their mRNA. 1) Untreated control, 2) Cells treated with Ionomycin 1 h previously, 3) Cells, in which Sp1 is repressed with RNA interference, and 4) Cells, stimulated with siRNA for Sp1 and additionally with Ionomycin for 1 h. The relative mRNA expression of c-Fos is determined by means of qRT-PCR. Data are presented as mean ± SD. The t-test was used for statistical evaluation of the data. P values of < 0.05 were considered statistically significant

To confirm the result of the expression profile analysis, we independently analyzed the mRNA level of c-Fos by means of quantitative RT-PCR **(**Fig. 2**)**. In this analysis, the activity of c-Fos in an untreated control group was normalized to the value of 100, and the influence of the regulatory change is shown as the x-fold increase or decrease of this control. The untreated control group showed significantly (4.5-fold) increased expression of c-Fos after the administration of Ionomycin (band 2). Repression of Sp1 with RNAi significantly influenced the effect of Ionomycin by Sp1 inhibition (band 4).

### Knock-down of the interaction partners NFATc2 and Sp1 with RNAi technology reduced cell proliferation

**Fig. 3:**
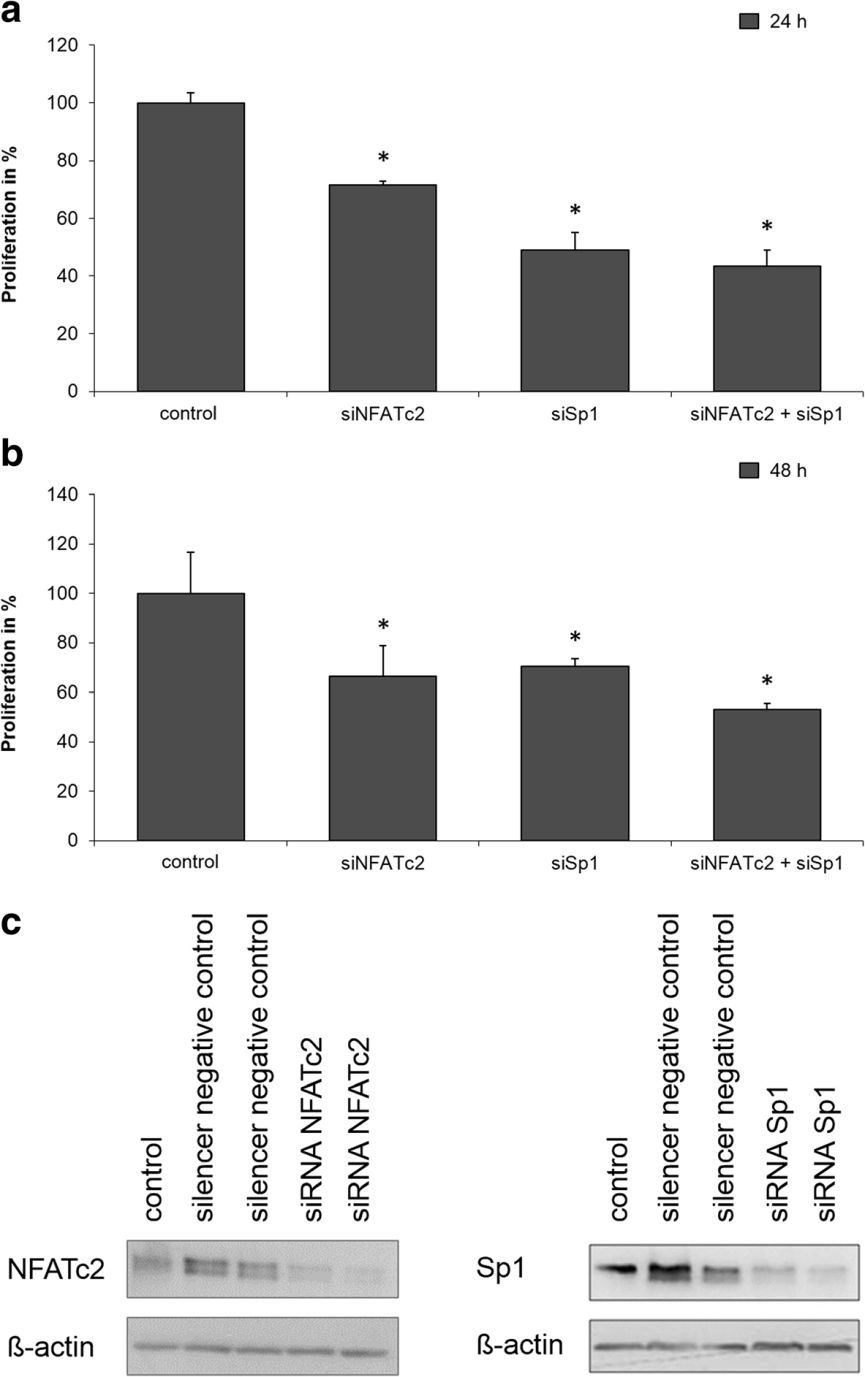
**a**, **b**, and **c**: Influence of NFATc2 and Sp1 on cell proliferation. PaTu 8988 t cells are transiently transfected with control-siRNA, siNFATc2, and/or siSp1 and stimulated with serum for 24 h (Fig. 3**a**) or 48 h (Fig. 3**b**). Fig. 3**c** shows the knockdown efficiency of the used siRNA

The impact of the interaction between NFATc2 and Sp1 on cell proliferation was investigated by means of a thymidine incorporation assay (Fig. 3). For this purpose, pancreatic cancer cells were transiently transfected with ‘Silencer Negative Control’-siRNA or a silencer-RNA sequence for NFATc2 or Sp1, or both. Proliferation was measured by means of radioactive thymidine incorporation into the cell in the beta counter. The activity of an untreated control group was normalized to the value of 100, and the influence of the regulatory change is shown as the x-fold increase or decrease of this control.

Basal proliferative activity of the cell was visible in band 1. Transfection of siNFATc2 (band 2) reduced proliferation to 71% after 24 h (Fig. 3a) and to 67% after 48 h (Fig. 3b), whereas transfection of siSp1 decreased proliferation to 49% (Fig. 3a) and 70% respectively (Fig. 3b). Inhibition of both transcription factors by siRNA knock-down (band 4) yielded a further but insignificant reduction in cell proliferation to 43% after 24 h and 53% after 48 h. Fig. 3c shows the knockdown efficiency of the siRNA used.

### Physical interaction between NFATc2 and the deletion mutant Sp1 N-terminus

**Fig. 4:**
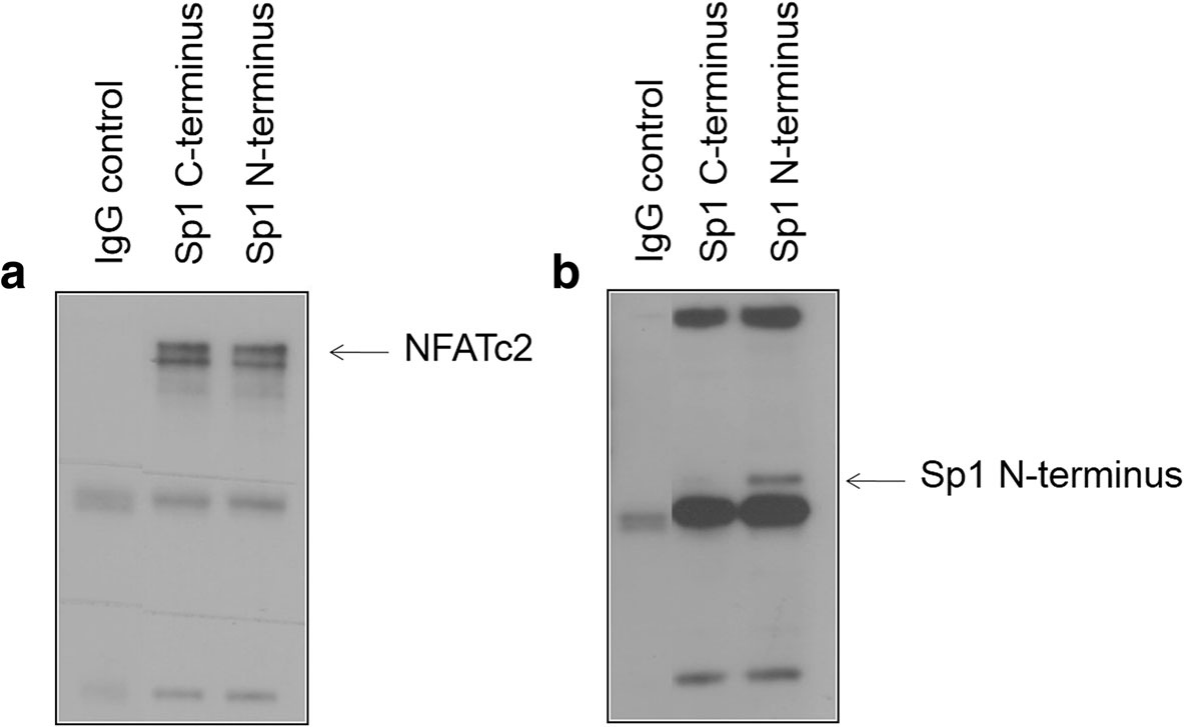
**a** and **b**: NFATc2 interacts with the N-terminus of Sp1. The deletion mutants FLAG-Sp1 C-terminus and FLAG-Sp1- N-terminus are over-expressed into the cell. NFATc2 proteins are immunoprecipitated via an antibody and Sp1 binding to NFATc2 is shown in Western blot analysis by means of an anti-FLAG antibody

Immunoprecipitation tests were conducted to investigate which domain of the Sp1 protein mediates interaction with NFATc2. For this purpose, the pancreatic cancer cell line PaTu 8988 t was transiently transfected with the effector plasmids of the Sp1 deletion mutants FLAG Sp1 C-terminus (zinc finger domain) and FLAG Sp1 N-terminus. NFATc2 was precipitated with agarose and NFATc2 antibodies and evaluated by means of Western blot analysis (Fig. 4a). The two bands of NFATc2 show the different stages of phosphorylization of the protein. Co-immunoprecipitated Sp1 deletion mutants were shown using anti-FLAG antibodies (Fig. 4b). The signal for Sp1 N-terminus was rather strong, but no signal could be detected for Sp1 C-terminus.

### Functional interaction between NFATc2 and Sp1 N-terminus in the dual luciferase assay

**Fig. 5:**
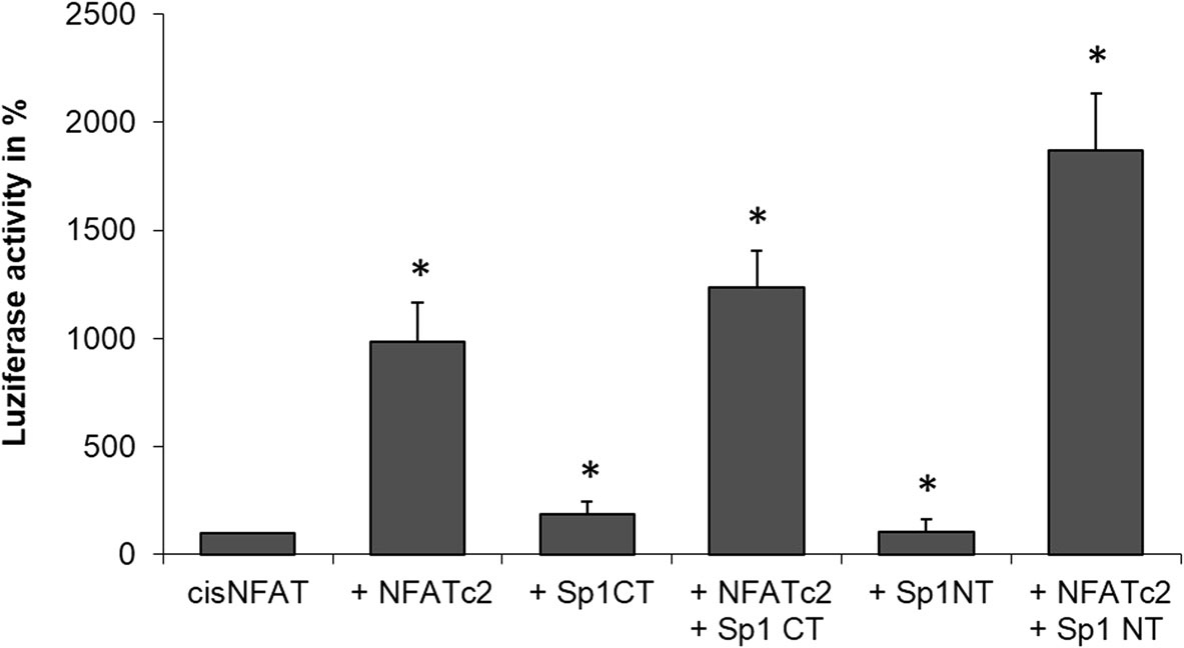
Functional interaction between NFATc2 and Sp1 in the dual luciferase assay. Dual luciferase assay by using the artificial NFAT-responsive reporter-promotor construct cisNFAT-Luc and the effector plasmids NFATc2, Sp1-CT, and Sp1-NT. Cells are harvested after 24 h, and promoter activation is determined by measuring light emission. Data are presented as mean ± SD. The t-test was used for statistical evaluation of the data. P values of < 0.05 were considered statistically significant

After having shown the physical interaction between NFATc2 and Sp1 N-terminus in immunoprecipitation tests, we investigated their functional interaction by means of the dual luciferase assay **(**Fig. 5**)**. After transient transfection of the artificial NFAT-responsive reporter-promotor-construct cisNFAT Luc as well as the effector plasmids NFATc2, Sp1 C-terminus, and Sp1 N-terminus into the cell, the emitting light was quantified with a luminometer. Basal activity of the promotor constructs cisNFAT Luc was normalized to a value of 100 followed by the assessment of the influence of the regulatory change.

Band 1 shows the basic basal activity of the promotors, whose effectiveness may be 10-fold increased by the transcription factor NFATc2 (band 2). Cell transfection only with Sp1 C-terminus or Sp1 N-terminus but not NFATc2 did not or just very slightly increase light emission (band 3 and 5). Co-transfection of both interaction partners only yielded a slight increase in band 4, but doubled light emission or promotor activity in band 6 in comparison to the sole transfection of NFATc2.

In summary, immunoprecipitation tests could confirm the physical interaction between NFATc2 and Sp1 N-terminus at the functional level.

## Discussion

Binding partners are a decisive factor in the specificity of transcription factors in cells. Recent investigations have shown the important role of the oncogenic transcription factor Sp1 in the transcriptional activity of NFATc2 in pancreatic cancer [13], in which interaction between binding partners occurs within the cell. However, the role of this interaction in pancreatic cancer is still unclear. In the current study, several target genes of NFATc2 were identified by means of expression profile analysis, such as the proto-oncogene c-Fos, the tumor necrosis factor TNF-alpha, the molecules APAF1, ATM, BCL2, BRCA1 and TNFRSF25 involved in apoptosis, the adhesion molecules integrin alpha-2 and integrin beta-3, the metastasis suppressor gene MTSS1, as well as phosphoinositide-3-kinase PI3K.

The tumor necrosis factor TNF-alpha takes a pivotal position amongst cytokines. TNF-alpha is a pleiotropic cytokine with a wide range of predominantly proinflammatory effects [16]. An anti-tumor effect has been observed in patients with carcinoma who developed an infectious disease, and this effect lasted over the entire course of the infectious disease until remission [17]. Furthermore, deregulated expression of TNF-alpha in the micro-environment of a tumor seems to promote tumoral invasion and migration and subsequently metastasis [18]. In experimental animal studies, treatment of ductal adenocarcinoma with TNF-alpha significantly facilitated tumor growth and metastasis [19], whereas treatment with infliximab and etanercept reduced tumor growth, for instance of liver metastases [20]. NFAT-dependent TNF-alpha expression in lymphocytes has already been described in the literature [21]. In this study, this regulation could also been shown in pancreatic cancer cells. Because Sp1 inhibition remarkably reduces the effect of Ionomycin, the DNA-binding activity of Sp1 also seems to play a role in NFATc2-mediated regulation.

Sp1 dependence may also be observed in c-Fos, a protein belonging to the bHLH-Zip protein family. C-Fos was first described by Finkel et al. in the 1970s, who managed to isolate retroviruses from bone tumors. The actual transforming insert has been termed Fos-oncogene [22]. In the following years, the cellular equivalent c-Fos was described for various types of tumors, and the protein involved in neoplastic transformation could be identified [23]. Transgenic mice that over-express this molecule develop osteosarcoma and chondrosarcoma. C-Fos causes high over-expression of cyclin D1 in osteoblasts and chondrocytes, and this over-expression results in the uncontrolled multiplication of cells [24]. Previous studies have found increased mRNA and protein expression of c-Fos in the majority of pancreatic cancer cells [25, 26]. Such induction has also been described for other types of tumors in which expression of c-Fos results in neoplastic transformation [23]. Saez et al. have shown the important role of c-Fos in the progression of malignant skin tumors [27]. Immunohistochemical examinations and in-situ hybridization studies have described the over-expression of c-Fos in esophagus carcinoma. The transcription factor c-Fos has been found in 66% of dysplasia and in 53% of squamous cell carcinoma but only in less than 5% of normal esophageal cellular tissue [28]. In contrast to c-Fos and TNF-alpha in which the effects of Ionomycin and Sp1 are pooled, the adhesion molecule ITGB3 has an antagonizing effect on mRNA expression and is suppressed by NFATc2 during treatment with Ionomycin.

Integrins are transmembrane, heterodimeric glycoproteins that consist of an alpha subunit and a beta subunit. These glycoproteins bind to various proteins of the extracellular matrix and mediate bidirectional signal transduction [29]. Integrins can be differentiated into 25 different subunits: 18 alpha and 8 beta subunits [30]. The occurrence of integrins is usually limited in time and restricted to the surface of cell membranes. The presence or absence of integrins has a strong impact on the growth, local invasion, destruction, and metastasis of malignant tumors [31]. The NFAT-dependent regulation of integrins was already described by Jauliac et al. in 2002 [32]. Because of the bipolar behavior of Sp1 that has already been described several times in the literature [33], NFATc2 und Sp1 may be assumed to mutually regulate the expression of ITGB3. Here, Sp1 acts as a transcriptional repressor of NFATcs2 in the respective GC box of the promoter.

In summary, data obtained from the expression profile analysis and the qRT-PCR examinations in the current study showed Sp1-dependent regulation of the promoters of c-Fos, TNF-alpha, and ITGB3. The effect of this regulation on pancreatic cancer cells was determined by means of a thymidine incorporation assay. Sole repression of the transcription factors resulted in a decrease by about 40–50%, and this percentage was only insignificantly diminished by mutual inhibition. One possible interpretation is that the binding partners NFATc2 and Sp1 interact and mutually regulate target genes dependent on cellular growth. Loss of just one transcription factor impedes oncogenic complex formation and expression of cell cycle-regulating genes. Therefore, one possible therapeutic approach would be inhibiting such interaction in the sense of ‘targeted therapy’ in pancreatic cancer cells. Modern therapeutic approaches already target efficient modulations of specific signaling and transcription pathways of individual factors [4]. This way, calcineurin-induced activation of NFAT may be inhibited by administrating the immunosuppressant Cyclosporin A that irreversibly binds to the catalytic domain of calcineurin, thus consecutively inhibiting the dephosphorylization of NFAT [34]. A further example is the peptide VIVIT that disrupts the interaction between calcineurin and NFAT, thus blocking the dephosphorylization of NFAT and impeding nuclear translocation [35]. Tolfenaminic acid, a non-steroidal anti-inflammatory drug, activates the degradation of Sp1, Sp3, and Sp4 and reduces expression of the vascular endothelial growth factor, hereby decreasing tumoral growth and metastasis [36]. Furthermore, some drugs inhibit specific Sp1-dependent transactivation [37], for instance oligonucleotides, peptide-nucleic acid, and DNA chimaeras. Other drugs such as mithramycin disrupt the binding of Sp-protein to the DNA [38], whereas agents such as the Cox-2 inhibitor increase Sp-protein degradation [36].

At present, several inhibitors are being investigated in the context of preclinical studies or have already been established in clinical practice [39]. A possible new approach to treating pancreatic cancer is inhibiting the interaction between NFATc2 and Sp1. As analyzed in the current study, it is essential to know the domain of the interaction between Sp1 and NFATc2 in the protein.

All proteins of Sp-like transcription factors show similar structural domains: the C-terminus has three zinc fingers, DNA-binding domains, a nuclear localization sequence (NLS), as well as a transcriptional-regulatory domain [40]. Zinc finger domains consist of 81 amino acids of the Cys_2_His_2_ type [41]; here, 4 cysteines or histidines act as ligands that form a zinc ion, so that the amino acid chains are reciprocally arranged to one another [42]. The N-terminus contains the activation domains Sp1 to Sp4 from glutamine-rich regions adjacent to serine-threonine-rich domains. Such threonine-rich domains are post-translationally modified by glycosylation on several threonine residues [43] as well as by phosphorylation and glycosylation on serine residues [44]. These domains play a pivotal role in the regulation of Sp1. Transcriptional activation is initiated in glutamine-rich subsections as well as in the D-domain (C-terminus), which has a synergistic effect due to the formation of multimers from two Sp1 molecules bound to the DNA as well as the protein-protein interaction of Sp1 molecules [45]. Sp1 and Sp3 also contain an inhibitory domain located at the N-terminus and the C-terminus [46].

Sp1 and other transcription factors of this family are primarily marked by their bipolar behavior [33]. Their function as an activator or repressor of transcription probably depends on the respective promotor or the respective transcription partner [47]. The domain responsible for this property is still unclear. Literature reports have described interaction with binding partners of the zinc fingers domains as well as the activating domains of the N-terminus of Sp1 [42]. Lee et al. hypothesized that the zinc finger domains of Sp1 are responsible for the interaction with other regulatory proteins because of their highly conservative amino acid sequence, which is assumed to act as a positive and negative regulator in the regulation of Sp1-DNA binding [45]. As shown in the structural analysis, the DNA-binding domains of Sp1, Sp3, and Sp4 are highly homologous in contrast to Sp2 [48]. Thus, it is not surprising that Sp1, Sp3, and Sp4 are able to bind to the classic Sp1-binding site of the GC box in the same way. Sp2, however, binds to a GT-rich element within the T-cell antigen receptor because of the change of amino acids from histidine to leucine at the first zinc finger [14]. Despite the pronounced homology between Sp1, Sp3, and Sp4, Sp1 has the highest activation potential. In contrast, Sp4 has less activation potential than Sp1, and Sp3 often has a suppressing effect on transcription [49]. On the other hand, the repressing functions of Sp1 and Sp3 are attributed to the inhibiting domains at the N-terminus but not in the zinc finger region [45].

The current study shows the interaction between the transcription factor NFATc2 and the N-terminal domain of Sp1 in pancreatic cancer cells. The functional relevance was shown by means of luciferase assays. Sp1 increases activity of NFATc2 at the NFAT-responsive promoter. One possible explanation may be the activating serine-threonine-rich and glutamine-rich domains of Sp1 that increase the transcription and function of NFATc2 in pancreatic cancer.

## Conclusions

The regulation of promotors in the context of transcription is a very complex process because of the involvement of several proteins that – as transcription factors or co-factors – regulate promotor activity as required and control cell function. In this respect, NFATc2 and Sp1 seem to play a key role in the progression of pancreatic cancer. The regulating binding partners interact within the cells and have a carcinogenic effect via transcriptional modification. Inhibitors that selectively inhibit the activity of this interaction may represent a novel approach to developing new therapeutic options for this aggressive type of tumor. Further studies are required to identify underlying mechanisms and examine their clinical importance in treating pancreatic cancer.

## Additional file

Additional file 1: Table S1. The gene using for the quantitative real-time polymerase chain reaction analysis and gene expression analysis. (DOCX 18 kb)

## Abbreviations

APAF1: Apoptotic protease activating factor 1
ATM: Ataxia telangiectasia
BCL2: B-cell lymphoma 2
BRCA1: Breast cancer 1.
ITGA_2_: Integrin alpha 2
ITGB_3_: Integrin beta 3
MTSS1: Metastasis suppressor gene 1
NFAT: Nuclear factors of activated T-cells
PanIN: Pancreatic intraepithal neoplasia
PI3K: Phosphoinositide-3-kinase
siRNA: Small interfering RNA
Sp1: Specificity protein 1
Sp1-CT: Sp1 Carboxyl-Terminus
Sp1-NT: Sp1 Amino-Terminus
TNF alpha: Tumor necrosis factor alpha
TNF beta: Tumor necrosis factor beta
TNFRSF25: TNF Receptor Family Member 25
