# Supplementary material for: The active role of the transcription factor Sp1 in NFATc2-mediated gene regulation in pancreatic cancer
Source: BMC Biochem. 2019 Jan 29;20:2. doi: 10.1186/s12858-019-0105-4 (PMC6352339; doi:10.1186/s12858-019-0105-4)
Supplement: Supplementary file 1 — The gene using for the quantitative real-time polymerase chain reaction analysis and gene expression analysis. (DOCX 18 kb) [file 12858_2019_105_MOESM1_ESM.docx]

*Supplemental Table S1:*

| *AKT1* | *CDC25A* | *FGFR2* | *ITGAV* | *MTA1* | *PLAUR* | *TGFBR1* | *B2M* |
| --- | --- | --- | --- | --- | --- | --- | --- |
| *ANGPT1* | *CDK2* | *FOS* | *ITGB1* | *MTA2* | *PNN* | *THBS1* | *HPRT1* |
| *ANGPT2* | *CDK4* | *GZMA* | *ITGB3* | *MTSS1* | *RAF1* | *TIMP1* | *RPL13A* |
| *APAF1* | *CDKN1A* | *HTATIP2* | *ITGB5* | *MYC* | *RB1* | *TIMP3* | *GAPDH* |
| *ATM* | *CDKN2A* | *IFNA1* | *JUN* | *NFKB1* | *S100A4* | *TNF* | *ACTB* |
| *BAD* | *CFLAR* | *IFNB1* | *MAP2K1* | *NFKBIA* | *SERPINB5* | *TNFRSF10B* |  |
| *BAX* | *CHEK2* | *IGF1* | *MCAM* | *NME1* | *SERPINE1* | *TNFRSF1A* |  |
| *BCL2* | *COL18A1* | *IL8* | *MDM2* | *NME4* | *SNCG* | *TNFRSF25* |  |
| *BCL2L1* | *E2F1* | *ITGA1* | *MET* | *PDGFA* | *SYK* | *TP53* |  |
| *BRCA1* | *ERBB2* | *ITGA2* | *MMP1* | *PDGFB* | *TEK* | *TWIST1* |  |
| *CASP8* | *ETS2* | *ITGA3* | *MMP2* | *PIK3R1* | *TERT* | *EPDR1* |  |
| *CCNE1* | *FAS* | *ITGA4* | *MMP9* | *PLAU* | *TGFB1* | *VEGFA* |  |
